# Low muscle mass is an independent risk factor for postoperative blood transfusion in total knee arthroplasty: a retrospective, propensity score-matched cohort study

**DOI:** 10.1186/s12877-022-02903-0

**Published:** 2022-03-17

**Authors:** Doohyun Hwang, Hyuk-Soo Han, Myung Chul Lee, Du Hyun Ro

**Affiliations:** 1 Department of Orthopedic Surgery, Seoul National University College of Medicine, 103 Daehak-ro, Jongno-gu Seoul, Republic of Korea; 2 Department of Orthopedic Surgery, Seoul National University Hospital, 101 Daehak-ro, Jongno-gu, Seoul, Republic of Korea; 3 CONNECTEVE Co., Ltd., 101 Daehak-ro, Jongno-gu Seoul, Republic of Korea

**Keywords:** Sarcopenia, Total knee arthroplasty, Transfusion, Bioelectrical impedance analysis, Complication, Skeletal muscle index

## Abstract

**Background:**

Sarcopenia, an age-related loss of skeletal muscle mass and function, is correlated with adverse outcomes after some surgeries. This study examined the characteristics of sarcopenic patients undergoing primary total knee arthroplasty (TKA), and identified low muscle mass as an independent risk factor for postoperative TKA complications.

**Methods:**

A retrospective cohort study examined 452 patients who underwent TKA. The skeletal muscle index (SMI) was obtained via bioelectrical impedance analysis (BIA), along with demographics, the Charlson Comorbidity Index, and medication, laboratory and operative data for 2018–2021. Patients were categorized into normal (*n =* 417) and sarcopenic (*n =* 35) groups using the SMI cut-off suggested by the Asian Working Group for Sarcopenia 2019 (males, < 7.0 kg/m^2^; females, < 5.7 kg/m^2^). Three postoperative complications were analysed: blood transfusion, delirium, and acute kidney injury (AKI). Baseline characteristics were propensity score-matched to address potential bias and confounding factors.

**Results:**

The proportion of sarcopenic patients in primary TKA was 7.7% (35/452). The sarcopenic group had a lower preoperative haemoglobin (12.18 ± 1.20 vs. 13.04 ± 1.73 g/dL, *p* = 0.004) and total protein (6.73 ± 0.42 vs. 7.06 ± 0.44 mg/dL, *p* = 0.001). Propensity scoring matching and logistic regression showed that more patients in the sarcopenic group received postoperative blood transfusions (OR = 6.60, 95% CI: 1.57–45.5, *p* = 0.021); there was no significant difference in AKI or delirium. Univariate receiver operating characteristic curve analysis of the propensity-matched group, to determine the predictive value of SMI for postoperative transfusion, gave an AUC of 0.797 (0.633–0.96) and SMI cut-off of 5.6 kg/m^2^.

**Conclusions:**

Low muscle mass determined by BIA was an independent risk factor for postoperative transfusion in TKA. Multifrequency BIA can serve as a screening tool for sarcopenia that may influence the orthopaedic decision-making process or treatment planning in patients with sarcopenia undergoing primary TKA.

**Level of evidence:**

III, retrospective cohort study.

**Supplementary Information:**

The online version contains supplementary material available at 10.1186/s12877-022-02903-0.

## Background

Total knee arthroplasty (TKA) is effective for treating severe degenerative arthritis of the knee, relieving pain and restoring mobility [1, 2]. TKA greatly enhances social and physical functioning, and thus the overall quality of life. As the population ages, the demand for TKA is increasing rapidly [3–5]. Despite its benefits, TKA involves substantial blood loss and approximately one-third of patients receive postoperative transfusions [6, 7]. Allogeneic transfusion is necessary for hemodynamic stability, but has side effects such as an increased risk of deep vein thrombosis, deep surgical site infection, and mortality, which adversely affect patient outcomes [8–12].

As the demand for TKA increases, patient selection and an understanding of the risk factors for postoperative transfusion should be emphasized. Many studies have evaluated the correlations between risk factors and complications of TKA, including postoperative blood transfusion, yet few studies have explored the effects of sarcopenia on complications of TKA [11, 13–15].

Sarcopenia was first characterized as an age-related loss of skeletal muscle mass [16]. As our understanding of sarcopenia has improved, it has been defined in various ways and attracted increasing academic interest [16–24]. The European Working Group on Sarcopenia in Older People defines sarcopenia as a “syndrome of progressive and generalized loss of skeletal muscle mass and strength, with a risk of adverse outcomes such as physical disability, poor quality of life and death” [25]. The reported prevalence of sarcopenia varies widely depending on the study population, and the prevalence in patients undergoing orthopaedic surgery appears to be higher than in the general population. Several studies have demonstrated that sarcopenia independently predicts adverse outcomes in patients undergoing general surgery, but there have been few reports on the impact of sarcopenia on orthopaedic surgery, including TKA [26, 27].

One validated method for assessing skeletal muscle mass is multifrequency bioelectrical impedance analysis (BIA) [21, 25]. This uses a combination of low and high frequencies of alternating current to calculate intercellular, extracellular, and total body water by measuring impedance (tissue resistance and reactance) as current passes through the body [28]. It is an attractive method for clinically identifying sarcopenic patients because it is affordable, non-invasive and can be completed in minutes [19]. BIA has been used extensively to assess sarcopenia in oncology patients [29]. It appears even more suitable for evaluating sarcopenia in orthopaedics, because radiological assessments such as magnetic resonance imaging, computed tomography, and dual-energy X-ray absorptiometry (DEXA) are not always available for these patients.

Although sarcopenia is attracting increasing attention, few orthopaedic studies have examined its impact, even when it is associated with increased morbidity and mortality in several surgical fields. Therefore, this study evaluated the impact of low muscle mass in patients undergoing TKA. Although the operational definition of sarcopenia requires an assessment of both the muscle mass and the muscle strength, we have focused on the quantitative determination of muscle mass, as it is the confirmatory diagnostic criterion for sarcopenia. We hypothesized that low muscle mass, as measured by BIA, correlates with an increased risk of postoperative blood transfusion, and that muscle mass quantity can predict blood transfusion after TKA. This study examined the characteristics of sarcopenic patients undergoing primary TKA, and evaluated the correlation between low muscle mass and blood transfusion after TKA.

## Methods

### Study subjects

After obtaining Institutional Review Board approval (IRB No. 1806–185-961), we conducted a retrospective, single-centre study. From May 2018 to April 2021, patients scheduled for primary TKA to treat degenerative knee arthritis were enrolled after providing informed consent. Subjects with adequate preoperative BIA, as determined using the InBody S10 device (InBody Co. Ltd., Seoul, Korea) were reviewed. Initially, 633 patients were enrolled. Patients who underwent simultaneous bilateral TKA (*n =* 5) or had inadequate tissue hydration (extracellular water [ECW] ratio > 0.4; *n =* 175) or severe obesity (body mass index [BMI] > 35 kg/m^2^; *n =* 1) were excluded [19, 21, 30], such that 452 patients (60 males, 392 females) were included in the final analysis.

### Operative technique and rehabilitation after TKA

Antiplatelet agents, including aspirin, clopidogrel, warfarin, heparin, and factor Xa inhibitors, were discontinued 1 week before surgery. The primary TKAs were performed in the identical manner. After midline skin incision, arthrotomy was performed with a parapatellar approach. An intramedullary guide was used to cut the femur and an extramedullary guide was used to cut the tibia. The intramedullary femoral canal was sealed with an autologous bone plug, with all implants being fixed with bone cement. After suturing the joint capsule, 1 g of tranexamic acid (TXA) was administered, unless contraindicated. All patients followed the same rehabilitation protocol, including full weight bearing gait and continuous passive motion (CPM) beginning 1 day after surgery. Ambulation was allowed 6 h after surgery [11, 31].

### Data collection

All data were collected from the institutional electronic medical records. Low muscle mass, referred to herein as “sarcopenic”, was defined using the cutoffs for the appendicular skeletal muscle index (SMI) suggested by the Asian Working Group for Sarcopenia 2019 (males, < 7.0 kg/m^2^; females, < 5.7 kg/m^2^) [21]. SMI is defined as the height-adjusted appendicular skeletal muscle mass (ASM) and is calculated via BIA. The baseline characteristics of the normal and sarcopenic groups were compared.

Patient demographics, comorbidities (modified Charlson Comorbidity Index [mCCI] and American Society of Anesthesiologists [ASA] score), medication history, and laboratory and operative data were collected. The mCCI was calculated by summing the weighted scores for individual comorbidities [32].

Three postoperative events were analysed: blood transfusion, delirium, and acute kidney injury (AKI). The transfusion group was defined as patients whose haemoglobin (Hb) levels dropped to less than 7 g/dL within 2 weeks after the first TKA [11]. Acute kidney injury was defined using the serum creatinine criteria of the Kidney Disease-Improving Global Outcomes group [33]. The Diagnostic and Statistical Manual of Mental Disorders, Fifth Edition defines the key feature of delirium as a disturbance in attention and awareness accompanied by an acutely fluctuating mental state [34].

### Statistical analysis

The statistical analyses were performed using RStudio for Windows (ver. 1.2.5033; RStudio, Boston, MA, USA). Nominal data are shown as percentages and were analysed with a two-sided Pearson’s χ^2^ test or Fisher’s exact test. Continuous data are shown as the mean ± SD and were analysed using Student’s *t-*test. Statistical significance was determined at *p* < 0.05. Simple binary logistic regression analysis was used to assess postoperative complications, and odds ratios (ORs) and 95% confidence intervals (CIs) were calculated.

To address potential bias and confounding factors, rigorous adjustment was conducted using 1:1 propensity score-matching (nearest neighbour matching). Propensity scores were estimated from multiple logistic regression analyses including all relevant covariates. The matching criteria were age at surgery, sex, BMI, mCCI, type of surgery, TXA use, preoperative Hb, preoperative platelets, and preoperative protein. After matching, 35 patients remained in each group (Table 1). Adjusted *p-*values and ORs were calculated for postoperative complications after propensity score-matching (Table 2).

**Table 1:** Baseline characteristics of the propensity score-matched sarcopenic and non-sarcopenic groups

Characteristics	Total population (*N =* 452)			Propensity-matched population (N = 70)		
	Low muscle mass			Low muscle mass		
	Yes (*n =* 35)	No (*n =* 417)	*p*-value	Yes (*n =* 35)	No (*n =* 35)	*p*-value
Number	35	417		35	35	
Age at surgery, years (SD)	74.5 (6.5)	70.5 (6.6)	0.001	74.51 (6.46)	74.19 (6.69)	0.684
Sex (%)						
Female	33 (94.3)	359 (86.1)	0.266	33 (94.3)	33 (94.3)	1
Male	2 (5.7)	58 (13.9)		2 (5.7)	2 (5.7)	
BMI, kg/m^2^ (SD)	23.9 (3.4)	26.7 (3.2)	< 0.001	23.9 (3.4)	24.1 (2.6)	0.691
mCCI			0.462			0.537
0	15 (52.9)	191 (45.8)		15 (42.9)	16 (45.7)	
1	12 (34.3)	94 (22.5)		12 (34.3)	8 (22.9)	
2	3 (8.6)	58 (13.9)		3 (8.6)	2 (5.7)	
≥ 3	5 (14.3)	74 (17.7)		5 (14.3)	9 (25.7)	
Type of surgery (%)			0.486			1
Unilateral (%)	20 (57.1)	211 (50.6)		20 (57.1)	20 (57.1)	
Bilateral (%)	15 (42.9)	206 (49.4)		15 (42.9)	15 (42.9)	
Tranexamic acid (%)	31 (88.6)	390 (93.5)	0.286	31 (88.6)	29 (82.9)	0.733
Haemoglobin, g/dL (SD)	12.2 (1.2)	13.0 (1.7)	0.004	12.2 (1.2)	12.2 (0.9)	0.830
Platelet count × /10^9^L (SD)	248.2 (54.1)	239.8 (57.7)	0.402	248.2 (54.1)	259.9 (60.5)	0.400
Total protein, mg/dL (SD)	6.7 (0.4)	7.1 (0.4)	< 0.001	6.7 (0.4)	6.8 (0.5)	0.753

Values are shown as the mean ± standard deviation or number (%). Statistical significance was set at *p* < 0.05

*BMI* Body mass index, *mCCI* modified Charlson Comorbidity Index

**Table 2:** Comparison of postoperative complications between the sarcopenic and non-sarcopenic groups

Characteristics	Total population (*N =* 452)				Propensity-matched population (*N =* 70)			
	Low muscle mass				Low muscle mass			
	Yes (*n =* 35)	No (*n =* 417)	*p*-value	OR (95% CI)	Yes (*n =* 35)	No (*n =* 35)	*p*-value	OR (95% CI)
Transfusion (%)	10 (28.6)	51 (12.2)	0.009	2.87 (1.25, 6.17)	10 (28.6)	2 (5.7)	0.021	6.60 (1.57, 45.5)
Acute Kidney Injury (%)	1 (2.9)	50 (12.0)	0.14	0.22 (0.01, 1.04)	1 (2.9)	6 (17.1)	0.079	0.14 (0.01, 0.90)
Delirium (%)	4 (11.4)	19 (4.6)	0.087	2.70 (0.75, 7.74)	4 (11.4)	2 (5.7)	0.4	2.13 (0.39, 16.1)

Values are presented as the mean ± standard deviation or number (%). Statistical significance was set at *p* < 0.05

## Results

The proportion of patients with low muscle mass who had undergone primary TKA was 7.7% (35/452). Patients in the normal and sarcopenic groups differed significantly in age at surgery, weight, height, BMI, preoperative Hb, and preoperative total protein (Table 1). Sarcopenic patients were older (74.5 ± 6.5 vs. 70.5 ± 6.6 years, *p* = 0.001) and had a lower BMI (23.9 ± 3.4 vs. 26.7 ± 3.2 kg/m^2^, *p* < 0.001), and lower preoperative levels of Hb (12.2 ± 1.2 vs. 13.0 ± 1.7 g/dL, *p* = 0.004) and total protein (6.7 ± 0.4 vs. 7.1 ± 0.4 mg/dL, *p* < 0.001).

Three postoperative complications were analysed using simple binary logistic regression. More patients in the sarcopenic group received postoperative blood transfusions (28.6 vs. 12.2%, OR = 2.87, 95% CI: 1.25–6.17, *p* = 0.009) (Table 2). However, there was no difference in the incidence of AKI or delirium.

After propensity score matching, no significant group difference was found in the demographic characteristics, surgical data, medications, or laboratory results of the two groups (Table 1). Binary logistic regression of the propensity score-matched groups showed that significantly more postoperative blood transfusions were administered to sarcopenic patients than to those without significant muscle loss (OR = 6.60, 95% CI: 1.57–45.5, *p* = 0.021) (Table 2).

**Fig. 1:**
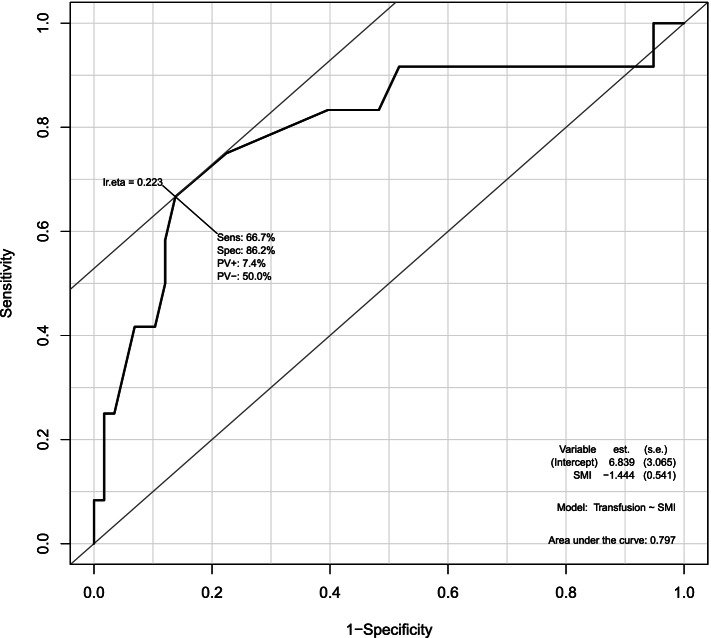
Receiver operating characteristic curve (ROC) for prediction of postoperative transfusion based on skeletal muscle index (SMI). The ROC analysis suggested an optimal SMI cut-off of 5.6 kg/m^2^, with an AUC value of 0.797 (0.633–0.96), sensitivity of 66.7%, and specificity of 86.2%

The correlation between the SMI, reflecting whole-body muscle quantity, and postoperative blood transfusion was also explored in the propensity score-matched groups. Univariate receiver operating characteristic (ROC) curve analysis was performed to determine the predictive accuracy of postoperative transfusion and the optimal cut-off value of the SMI. The ROC analysis suggested an SMI cut-off of 5.6 kg/m^2^, with an AUC value of 0.797 (0.633–0.96), sensitivity of 66.7%, and specificity of 86.2% (Fig. 1).

## Discussion

Sarcopenia is attracting attention as an independent predictor of postoperative morbidity and mortality. In arthroplasty, sarcopenia is associated with an increased risk of prosthetic infection after total hip or knee arthroplasty [35]. Patients with sarcopenia undergoing thoracolumbar spine surgery have an increased risk of postoperative complications and mortality, and significantly longer hospital stays [17]. Sarcopenia is also an independent risk factor for fragility fractures in all patients, and is responsible for the higher 1-year mortality rate of elderly sarcopenic patients with acetabular fractures (28.6% vs. 12.3%) [36, 37].

The correlations between sarcopenia and postoperative complications are becoming increasingly clear. Some studies have attempted to elucidate the correlation between sarcopenia and postoperative blood transfusion. Sarcopenia is thought to be associated with blood transfusions in head and neck cancer free-flap surgery [38]. Ardeljan et al. studied 90,438 patients who had undergone primary TKA; 16.7% of them had sarcopenia. The patients with sarcopenia had longer hospital stays and increased odds of falls, lower extremity fractures, reoperation, 2-year implant-related complications, surgery costs, and complications within 90 days, one of which was transfusion with blood products (0.47% vs. 0.13%, OR = 4.24, 95% CI: 3.09–5.82, *p* < 0.0001) [29]. Our results are similar, in that sarcopenic patients with low muscle mass had a higher risk of postoperative blood transfusion than those without sarcopenia (OR = 6.60, 95% CI: 1.57–45.5, *p* = 0.021), while there were no obvious correlations with postoperative AKI or delirium.

To the best of our knowledge, no study has focused on muscle mass quantity as an important predictor of postoperative transfusion. Furthermore, no study has used BIA to evaluate sarcopenia. Our results indicate a strong correlation between low muscle mass determined by BIA and postoperative transfusion, and thus that postoperative blood transfusions are more frequent in sarcopenic than non-sarcopenic patients (28.6 vs. 12.2%, OR = 2.87, 95% CI: 1.25–6.17, *p* = 0.009). After propensity-scored matching, low muscle mass continued to be a significant predictor of postoperative transfusion (28.6 vs. 5.7%, OR = 6.60, 95% CI: 1.57–45.5, *p* = 0.021). ROC curve analysis showed that the SMI, as a predictor of postoperative transfusion, had an area under the curve of 0.797, sensitivity of 66.7%, and specificity of 86.2%, illustrating that the SMI discriminates well between transfused and non-transfused patients.

The pathophysiology of the increased rate of transfusions in sarcopenic patients is unclear. However, it might be related to the role of skeletal muscle as a vascular reservoir, given its high capillary density compared to other soft tissues such as adipose tissue [38]. Because sarcopenic patients have lower skeletal muscle mass, their total blood volume is also reduced and these patients might be more susceptible to blood loss [39].

Cross-sectional studies have revealed that knee osteoarthritis is associated with declines in muscle mass and strength in the lower limbs as the patient adapts to a sedentary lifestyle and inactivity to avoid knee pain and stiffness [40]. Since the majority of patients undergoing TKA have end-stage osteoarthritis, TKA patients may have a markedly decreased lower limb muscle mass, especially on the side requiring TKA. This supports the idea that a patient undergoing TKA is prone to sarcopenic condition, leading to more frequent postoperative blood transfusions.

One limitation of this study was the questionable validity of using multifrequency BIA to determine muscle mass. Multifrequency BIA is widely accepted as a validated measure for assessing sarcopenia in European and Asian guidelines, and by the international research community studying cachexia [21, 25, 41]. BIA is considered to have high concurrent validity for muscle mass estimation in normally hydrated and non-severely obese patients, making it feasible for evaluating low lean muscle mass and diagnosing sarcopenia [19]. The accuracy of BIA depends heavily on the adequacy of tissue hydration, and it should be used carefully in morbidly obese or overhydrated patients, as it can lead to overestimation of fat-free mass. Therefore, in our study, patients with inadequate tissue hydration and severe obesity were excluded to minimize the likelihood of muscle mass overestimation [30]. However, muscle mass may still have been somewhat overestimated, which might have led to the comparatively low proportion of low muscle mass compared to previous studies. Although there are consensus cut-off values for determining sarcopenia in Europe and Asia, populations of the same race may differ, so there is a need for population-specific cut-offs for determining sarcopenia [42, 43].

We do not claim that BIA is a perfect tool for assessing sarcopenia; our aim was only to assess the use of BIA for screening sarcopenic patients undergoing orthopaedic surgery, including TKA. For preoperative risk stratification of TKA patients, prospective studies including both muscle quantity and functional tests have to be conducted to delineate clear correlations between sarcopenia and adverse outcome of TKA patients. Functional testing may include gait speed, muscle grip strength, the get-up-and-go test, and peak expiratory flow [21]. Multifrequency BIA can serve as a screening tool for sarcopenia, as described here. Diagnoses of patients with low muscle mass at screening can then be confirmed, if necessary, via DEXA, which is the gold standard for quantifying muscle mass [25]. A long-term, retrospective review of the clinical outcomes of the sarcopenic patients in this cohort who underwent primary TKA, including postoperative pain, physical activity, and level of satisfaction, is ongoing. We hope to present the conclusions of this review in the near future.

Sarcopenia is a modifiable risk factor that can be prevented and managed [44, 45]. Evidence-based guidelines published by the American Medical Directors Association suggest that adequate protein intake and resistance exercises can enhance muscle strength [46]. Further studies should examine how to minimize the surgical complications of joint arthroplasty by identifying and adjusting modifiable risk factors, such as sarcopenia.

## Conclusion

In conclusion, among our patients undergoing primary TKA, 7.7% were sarcopenic, and more of these patients received postoperative blood transfusions than non-sarcopenic patients. Orthopaedic surgeons should be aware of this, as it could influence the decision-making process or treatment plan of patients with sarcopenia undergoing primary TKA.

## Supplementary Information

**Additional file 1: Supplementary Table 1.** Baseline characteristics of sarcopenic and non-sarcopenic patients. **Supplementary Table 2.** Laboratory data of sarcopenic and non-sarcopenic patients.

## Data Availability

All data generated or analysed during this study are included in this published article and its supplementary information files.

## Abbreviations

TKA: Total knee arthroplasty
BIA: Bioelectrical impedance analysis
AKI: Acute kidney injury
SMI: Skeletal muscle index
AUC: Area under the curve
DEXA: Dual-energy X-ray absorptiometry
BMI: Body mass index
TXA: tranexamic acid
mCCI: modified Charlson comorbidity index
ASA: American Society of Anesthesiologists
ROC: Receiver operating characteristic
