# Supplementary material for: Low muscle mass is an independent risk factor for postoperative blood transfusion in total knee arthroplasty: a retrospective, propensity score-matched cohort study
Source: BMC Geriatr. 2022 Mar 17;22:218. doi: 10.1186/s12877-022-02903-0 (PMC8928693; doi:10.1186/s12877-022-02903-0)
Supplement: Supplementary file 1 — Additional file 1: Supplementary Table 1. Baseline characteristics of sarcopenic and non-sarcopenic patients. Supplementary Table 2. Laboratory data of sarcopenic and non-sarcopenic patients. [file 12877_2022_2903_MOESM1_ESM.docx]

Supplementary Table 1. Baseline characteristics of sarcopenic and non-sarcopenic patients.

| **Characteristics** | | **All patients**  **(N=452)** | | **Sarcopenic**  **(N=35)** | | **Normal**  **(N=417)** | | ***p*-value** | | Missing data | |
| --- | --- | --- | --- | --- | --- | --- | --- | --- | --- | --- | --- |
| Demographics | |  | |  | |  | |  | |  | |
| Age at surgery, year (SD) | | 70.82 (6.66) | | 74.51 (6.46) | | 70.51 (6.59) | | 0.001 | |  | |
| Gender (%) | |  | |  | |  | |  | |  | |
| Female | | 392 (86.7) | | 33 (94.3) | | 359 (86.1) | | 0.266 | |  | |
| Male | | 60 (13.3) | | 2 (5.7) | | 58 (13.9) | |  | |  | |
| Height, cm (SD) | | 154 (6.70) | | 149 (5.82) | | 155 (6.61) | | <0.001 | |  | |
| Weight, kg (SD) | | 63.1 (9.23) | | 53.3 (8.38) | | 64.0 (8.82) | | <0.001 | |  | |
| BMI, kg/m^2^ (SD) | | 26.50 (3.26) | | 23.86 (3.36) | | 26.72 (3.16) | | <0.001 | |  | |
| Underweight (%) | | 4 (0.9) | | 3 (8.6) | | 1 (2.9) | |  | |  | |
| Normal (%) | | 142 (31.4) | | 17 (48.6) | | 125 (30.0) | |  | |  | |
| Overweight (%) | | 244 (54.0) | | 14 (40.0) | | 230 (55.2) | |  | |  | |
| Obese (%) | | 62 (13.7) | | 1 (2.9) | | 61 (14.6) | |  | |  | |
| Smoking (%) | | 8 (1.8) | | 0 (0) | | 8 (1.9) | | 1 | |  | |
| Alcohol (%) | | 32 (7.1) | | 0 (0) | | 32 (7.7) | | 0.159 | |  | |
| BIA-measured parameters | |  | |  | |  | |  | |  | |
| Skeletal muscle index, kg/m^2^ (SD) | | 7.28 (1.16) | | 5.45 (0.597) | | 7.43 (1.06) | | < 0.001 | |  | |
| Leg skeletal mass, right, kg (SD) | | 6.91 (1.71) | | 4.52 (0.776) | | 7.11 (1.61) | | < 0.001 | |  | |
| Leg skeletal mass, left, kg (SD) | | 6.77 (1.59) | | 4.55 (0.736) | | 6.96 (1.50) | | < 0.001 | |  | |
| Medication | |  | |  | |  | |  | |  | |
| Tranexamic acid (%) | | 421 (93.1) | | 31 (88.6) | | 390 (93.5) | | 0.286 | |  | |
| Antiplatelet (%) | | 420 (92.9) | | 34 (97.1) | | 386 (92.6) | | 0.497 | |  | |
| Anticoagulant (%) | | 88 (19.5) | | 7 (20.0) | | 81 (19.4) | | 1 | |  | |
| Dexamethasone (%) | | 442 (97.8) | | 34 (97.1) | | 408 (97.8) | | 0.557 | |  | |
| Celecoxib (%) | | 444 (98.2) | | 34 (97.1) | | 410 (98.3) | | 0.478 | |  | |
| Comorbidities | |  | |  | |  | |  | |  | |
| ASA class | |  | |  | |  | | 0.302 | |  | |
| 0 | | 2 ( 0.4) | | 0 ( 0.0) | | 2 ( 0.5) | |  | |  | |
| 1 | | 76 (17.0) | | 7 (20.6) | | 69 (16.7) | |  | |  | |
| 2 | | 343 (76.9) | | 23 (67.6) | | 320 (77.7) | |  | |  | |
| 3 | | 25 ( 5.6) | | 4 (11.8) | | 21 ( 5.1) | |  | |  | |
| mCCI | |  | |  | |  | | 0.462 | |  | |
| 0 | | 206 (45.6) | | 15 (52.9) | | 191 (45.8) | |  | |  | |
| 1 | | 106 (23.5) | | 12 (34.3) | | 94 (22.5) | |  | |  | |
| 2 | | 61 (13.5) | | 3 (8.6) | | 58 (13.9) | |  | |  | |
| ≥3 | | 79 (17.5) | | 5 (14.3) | | 74 (17.7) | |  | |  | |
| Hypertension (%) | | 170 (37.6) | | 8 (22.9) | | 162 (38.8) | | 0.070 | |  | |
| Diabetes mellitus (%) | | 89 (19.7) | | 11 (31.4) | | 78 (18.7) | | 0.078 | |  | |
| Asthma (%) | | 11 (2.4) | | 1 (2.9) | | 10 (2.4) | | 0.592 | |  | |
| COPD (%) | | 3 (0.7) | | 1 (2.9) | | 2 (0.5) | | 0.215 | |  | |
| Cardiovascular disease (%) | | 61 (13.5) | | 5 (14.3) | | 56 (13.4) | | 0.8 | |  | |
| Peripheral vascular disease (%) | | 78 (17.3) | | 3 (8.6) | | 75 (18.0) | | 0.241 | |  | |
| Cerebrovascular disease  (%) | | 53 (11.7) | | 5 (14.3) | | 48 (11.5) | |  | |  | |
| Comorbid degenerative  arthritis (%) | | 59 (13.1) | | 9 (25.7) | | 50 (12.0) | | 0.064 | |  | |
| Foot & ankle (%) | | 6 (1.3) | | 2 (5.7) | | 4 (1.0) | |  | |  | |
| Hand (%) | | 8 (1.8) | | 1 (2.9) | | 7 (1.7) | |  | |  | |
| Hip (%) | | 4 (0.9) | | 0 (0) | | 4 (1.0) | |  | |  | |
| Shoulder (%) | | 1 (0.2) | | 0 (0) | | 1 (0.2) | |  | |  | |
| Multiple (%) | | 40 (8.8) | | 6 (17.1) | | 34 (8.2) | |  | |  | |
| Comorbid degenerative  spondylosis (%) | | 166 (36.4) | | 12 (34.3) | | 154 (36.9) | | 0.899 | |  | |
| Lumbosacral (%) | | 147 (32.5) | | 12 (34.3) | | 135 (32.4) | |  | |  | |
| Cervical (%) | | 6 (1.3) | | 0 (0) | | 6 (1.4) | |  | |  | |
| Both (%) | | 13 (2.9) | | 0 (0) | | 13 (3.1) | |  | |  | |
| Osteoporosis (%) | |  | |  | |  | | 0.051 | | 193 | |
| Normal (%) | | 80 (30.9) | | 2 ( 9.5) | | 78 (32.8) | |  | |  | |
| Osteopenia (%) | | 129 (49.8) | | 12 (57.1) | | 117 (49.2) | |  | |  | |
| Osteoporosis (%) | | 50 (19.3) | | 7 (33.3) | | 43 (18.1) | |  | |  | |
| Liver disease (%) | | 46 (10.2) | | 3 (8.6) | | 43 (10.3) | | 1 | |  | |
| Renal disease (%) | | 20 (4.4) | | 2 (5.7) | | 18 (4.3) | | 0.662 | |  | |
| Thyroid disease (%) | | 60 (13.3) | | 4 (11.4) | | 56 (13.4) | | 1 | |  | |
| Rheumatoid disease (%) | | 17 (3.8) | | 1 (2.9) | | 16 (3.8) | | 1 | |  | |
| Malignancy (%) | | 49 (10.8) | | 3 (8.6) | | 46 (11.0) | | 1 | |  | |
| Surgery information | |  | |  | |  | |  | |  | |
| Type of surgery (%) | |  | |  | |  | | 0.404 | |  | |
| Unilateral, right (%) | | 102 (22.6) | | 11 (31.4) | | 91 (21.8) | |  | |  | |
| Unilateral, left (%) | | 129 (28.5) | | 9 (25.7) | | 120 (28.8) | |  | |  | |
| Bilateral, staged (%) | | 221 (48.9) | | 15 (42.9) | | 206 (49.4) | |  | |  | |
| Operation time, min (SD) | | 84.9 (22.0) | | 83.95 (16.64) | | 84.98 (22.42) | | 0.798 | |  | |
| Anesthesia | |  | |  | |  | | 0.469 | |  | |
| General anesthesia (%) | | 28 (6.2) | | 3 (8.6) | | 25 (6.0) | |  | |  | |
| Spinal anesthesia (%) | | 424 (83.8) | | 32 (91.4) | | 392 (94.0) | |  | |  | |

Values are shown as the mean ± standard deviation or number (%). Statistical significance was set at *p*<0.05.

ASA, American society of anesthesia; COPD, Chronic obstructive pulmonary disease

| **Characteristics** | **All patients**  **(N=452)** | **Sarcopenic**  **(N=35)** | **Normal**  **(N=417)** | ***p*-value** |
| --- | --- | --- | --- | --- |
| PT INR (SD) | 0.96 (0.06) | 0.95 (0.05) | 0.96 (0.06) | 0.495 |
| Hemoglobin, g/dL (SD) | 12.97 (1.71) | 12.18 (1.20) | 13.04 (1.73) | 0.004 |
| Platelet count × /10^9^L (SD) | 240.42 (57.37) | 248.23 (54.10) | 239.76 (57.65) | 0.402 |
| ESR, mm/h (SD) | 18.40 (13.07) | 15.51 (10.56) | 18.64 (13.24) | 0.174 |
| C-Reactive Protein, mg/dL  (SD) | 0.18 (0.45) | 0.10 (0.11) | 0.19 (0.46) | 0.248 |
| Albumin, g/dL (SD) | 4.23 (0.36) | 4.12 (0.28) | 4.23 (0.37) | 0.081 |
| Total Protein, mg/dL (SD) | 7.03 (0.44) | 6.73 (0.42) | 7.06 (0.44) | <0.001 |
| AST(GOT)^c^, mg/dL (SD) | 24.54 (13.41) | 22.06 (4.53) | 24.75 (13.89) | 0.255 |
| ALT(GPT)^d^, mg/dL (SD) | 22.17 (11.83) | 18.71 (7.98) | 22.46 (12.06) | 0.072 |
| Alkaline phosphatase,  mg/dL (SD) | 70.83 (22.38) | 66.51 (21.47) | 71.19 (22.44) | 0.235 |
| Total Bilirubin, mg/dL (SD) | 0.62 (0.24) | 0.62 (0.24) | 0.62 (0.25) | 0.87 |
| BUN, mg/dL (SD) | 18.43 (5.83) | 20.31 (10.08) | 18.27 (5.31) | 0.046 |
| Creatinine, mg/dL (SD) | 0.79 (0.24) | 0.80 (0.44) | 0.79 (0.21) | 0.769 |
| eGFR - MDRD (SD) | 79.24 (16.97) | 81.79 (24.28) | 79.03 (16.23) | 0.356 |
| eGFR – CKD EPI (SD) | 80.07 (14.47) | 78.68 (18.02) | 80.18 (14.15) | 0.556 |
| Sodium, mg/dL (SD) | 141.22 (1.92) | 141.51 (1.87) | 141.19 (1.93) | 0.339 |
| Potassium, mg/dL (SD) | 4.31 (0.41) | 4.35 (0.40) | 4.30 (0.42) | 0.534 |
| Chloride, mg/dL (SD) | 104.99 (2.38) | 105.09 (2.38) | 104.98 (2.38) | 0.798 |
| Total Calcium, mg/dL (SD) | 9.22 (0.42) | 9.27 (0.45) | 9.22 (0.42) | 0.473 |
| Phosphorus, mg/dL (SD) | 3.67 (0.50) | 3.67 (0.48) | 3.67 (0.50) | 0.985 |
| Uric acid, mg/dL (SD) | 4.82 (1.22) | 4.45 (1.31) | 4.85 (1.21) | 0.065 |
| Glucose, mg/dL (SD) | 124.18 (37.53) | 128.51 (42.17) | 123.82 (37.14) | 0.478 |
| HbA1c, mmol/mol (SD) | 5.98 (0.73) | 5.96 (0.59) | 5.98 (0.74) | 0.895 |

Supplementary Table 2. Laboratory data of sarcopenic and non-sarcopenic patients.

Values are shown as the mean ± standard deviation (SD). Statistical significance was set at *p*<0.05.

ESR, Erythrocyte Sedimentation Rate; BUN, Blood Urea Nitrogen

**SUPPLEMENTARY INFORMATION**

Additional file 1: Supplementary Table 1. Baseline characteristics of sarcopenic and non-sarcopenic patients. Supplementary Table 2. Laboratory data of sarcopenic and non-sarcopenic patients.
